# Diagnostic prediction of complex diseases using phase-only correlation based on virtual sample template

**DOI:** 10.1186/1471-2105-14-S8-S11

**Published:** 2013-05-09

**Authors:** Shu-Lin Wang, Yaping Fang, Jianwen Fang

**Affiliations:** 1 Applied Bioinformatics Laboratory, the University of Kansas, 2034 Becker Drive, Lawrence, KS 66047, USA

## Abstract

**Motivation:**

Complex diseases induce perturbations to interaction and regulation networks in living systems, resulting in dynamic equilibrium states that differ for different diseases and also normal states. Thus identifying gene expression patterns corresponding to different equilibrium states is of great benefit to the diagnosis and treatment of complex diseases. However, it remains a major challenge to deal with the high dimensionality and small size of available complex disease gene expression datasets currently used for discovering gene expression patterns.

**Results:**

Here we present a phase-only correlation (POC) based classification method for recognizing the type of complex diseases. First, a virtual sample template is constructed for each subclass by averaging all samples of each subclass in a training dataset. Then the label of a test sample is determined by measuring the similarity between the test sample and each template. This novel method can detect the similarity of overall patterns emerged from the differentially expressed genes or proteins while ignoring small mismatches.

**Conclusions:**

The experimental results obtained on seven publicly available complex disease datasets including microarray and protein array data demonstrate that the proposed POC-based disease classification method is effective and robust for diagnosing complex diseases with regard to the number of initially selected features, and its recognition accuracy is better than or comparable to other state-of-the-art machine learning methods. In addition, the proposed method does not require parameter tuning and data scaling, which can effectively reduce the occurrence of over-fitting and bias.

## Introduction

Classification and diagnostic prediction of complex diseases such as cancers and neuron-degeneration diseases using genomic or proteomic data can improve the quality of pathological diagnosis and help develop personalized treatment of these diseases [1]. Although great efforts have been exerted in this field, making early and precise diagnosis of complex diseases, followed through with effectively treating remains a great challenge. For example, the histological methods cannot precisely distinguish between the subtypes of some cancers [2] that the development of effective therapies depends on. The molecular mechanisms of many neuron-degeneration diseases such as Alzerheimer's (AD) and Parkinson's (PD) diseases are not fully understood and diagnosis of these diseases rely on medical history evaluation and the combination of physical and neurological assessments [3,4], often after irreversible brain damage or mental decline already occurs.

The rationale of classification and diagnostic prediction of complex diseases using genomic or proteomic data is based on the assumption that complex diseases induce perturbations to interaction and regulation networks of living systems, resulting in dynamic equilibrium states that differ for different diseases and also normal states. Thus identifying gene expression patterns corresponding to different equilibrium states is a key task to the success of these types of approaches. Many pattern recognition methods based on machine learning, such as *k*-nearest neighbor (KNN), support vector machines (SVM) [5-7], probabilistic neuron networks (PNN) [8-10], naive Bayes model (NBM) [11] and random forest (RF) [4,12], etc., have been extensively explored for the classification and diagnostic prediction of complex diseases [13]. Usually, these supervised learning methods are called model-based ones because a classification model needs to be constructed using a training set before it can be used to predict the label of a test sample. However, for the model-based methods, feature extraction and feature selection techniques play a vital role in improving the performance of complex disease classification due to the high-dimensionality and small sample size of GEP dataset.

An example of feature extraction methods is that independent component analysis (ICA) was used to extract independent components from GEP to reduce the dimensionality of sample [7,14,15]. Other feature extraction methods such as principal component analysis (PCA) [6], linear discriminant analysis (LDA) [16] and locally linear discriminant embedding (LLDE) [17] are also extensively applied to the dimensionality reduction of GEP. Although such methods can achieve satisfactory classification performance, there is weak biomedical interpreter and significance. An example of gene selection methods is that the Classification to Nearest Centroids (ClaNC) method for class-specific gene selection was proposed to determine a gene subset of given size that maximizes the classification accuracy [18]. Although such methods have biomedical meaning, there are a great number of gene subsets with the same predictive performance, which could lead to the selection arbitrariness of candidate gene subsets. In fact, each method has its drawbacks, and many factors such as normalization, small sample size, noisy data, improper evaluation methods, and too many model parameters can lead to the over-fitting of the constructed model, the bias of results and false discovery [19-22]. Even so, "microarrays remain a useful technology to address a wide array of biological problems and the optimal analysis of these data to extract meaningful results still pose many bioinformatics challenges." [23]. Therefore, with the increasing accumulation of GEP and protein microarray data, it is still necessary to design more effective and more biomedical methods to recognize complex disease type, which is also the requirement of clinical application.

For potential clinical applications, a candidate classification model should be evaluated for three aspects: accuracy, interpretability and practicality [18]. And a novel method should be measured up from three aspects. 1) A good model should be simple and have no or few parameters to be tuned. If parameters are necessary, the model should be robust with regard to the variation of these parameters. 2) The obtained model should achieve the best or near-optimal performance of disease classification as compared to the relevant state-of-the-art methods because there is no classification method that always outperforms all others in all circumstances [23,24]. 3) The obtained model should be obviously interpretable from biomedical perspective, which requires that the intrinsic signatures of sample set should be used as designing the classification model.

Previous studies suggest that each complex disease type or subtype corresponds to a dynamic equilibrium state of disease-induced genomic interaction and regulation network, and different samples at the same state are similar in gene expression profiles [25]. Thus analyzing the similarity level of gene expression profiles can be in principle used to distinguish different disease types or subtypes. A gene expression profile, which comprises the expression levels of numerous genes, can be likened to a digital image that consists of the luminance of pixels. In fact, both microarray and protein array data are originated from digital images. We therefore suggest that it is reasonable to apply some image processing methods to analyze genomic and proteomic data. Based on this idea, recently we successfully proposed two correlation filters based on tumor classification methods, namely, minimum average correlation energy (MACE) and optimal tradeoff synthetic discriminant function (OTSDF), to identify the overall pattern of differentially expressed genes (DEGs), corresponding to the tumor subtypes [26]. Although the two methods perform well in classifying tumor subtypes, they have some drawbacks: 1) The two methods are sensitive to the data scaling methods used to standardize the data; 2) although the template synthesized for each subtype in frequency domain space can be used to characterize the corresponding subtype, the biomedical significance of the synthesized template itself is not obvious enough. Thus it is highly desirable to explore other correlation methods which can recognize disease types well but without the weaknesses of the MACE and OTSDF-based disease classification methods.

Our further experiments indicate that phase-only correlation (POC) [27] may be such a method. Like the MACE and OTSDF filters, POC also utilizes a fast frequency domain approach to estimate the similarity degree between two samples. In recent years POC has also been extensively applied to image recognition [28,29] and identification of seismic events [30]. In this study, we present a novel POC-based method to complex disease classification based on virtual sample templates using genomic or proteomic data. First, we construct one template for each subclass on a training set. Sample matching can then be performed by cross-correlating a test sample with each template in training set using POC and analyzing the resulting correlation output. By comparing the peaks of correlation output, the test sample can be easily assigned to the class for which the template with the highest similarity to the test sample represents.

## Methods

### Complex disease datasets

Seven public available complex disease datasets are used to evaluate the proposed method in our experiments. They include the Leukemia1 [31], GSE29676 (http://www.ncbi.nlm.nih.gov) [3,4], Leukemia2 [32], small round blue cell tumor (SRBCT) [33], GSE5281 (http://www.ncbi.nlm.nih.gov) [34], colon tumor (Colon) [35], and GCM [36] datasets. The Leukemia1 dataset contains 72 samples from three subtypes or subclasses, i.e., MLL, AML and ALL. The GSE29676 dataset includes 50 Alzheimer's disease and 29 Parkinson's disease samples as well as 40 non-demented control samples. The Leukemia2 dataset contains 72 samples and 7,129 genes from three subclasses, i.e., AML, ALL-T and ALL-B. The GSE5281 dataset includes 71 normal samples and 87 Alzheimer samples. The SRBCT dataset consists of four subclasses, i.e., Ewing's sarcoma (EWS), Burkitt's (BL), Neuroblastoma (NB), and rhabdomyosarcoma (RMS). The GCM dataset consists of fourteen different tumor types. These datasets are summarized in Table 1.

**Table 1:** The summary of the seven complex disease datasets.

Datasets	Platform	#Samples	#Features	#Subclasses(*K*)
Leukemia1	Affy HGU95a	72	12,582	3
GSE29676	Invitrogen ProtoArray v5.0	119	9,480	3
Leukemia2	Affy HU6800	72	7,129	3
SRBCT	cDNA	83	2,308	4
GSE5281	Affy HG-U133	161	54,675	2
Colon	Affy HUM6000	62	2,000	2
GCM	Affy HU6800	190	16,063	14

Both protein and DNA microarray data can be represented with matrices. Thus we use DNA microarray data as an example to describe the design of our method. Let $G=\left\{g_{1},g_{2},\cdots \;,g_{N}\right\}$ denote a set of *N *genes, and $S=\left\{f_{1}\left(s\right),f_{2}\left(s\right),\cdots \;,f_{M}\left(s\right)\right\}$ denote a set containing M  samples, where $f_{m}\left(s\right)={\left(x_{m,1},\cdots x_{m,n},\cdots x_{m,N}\right)}^{\prime },1\leq m\leq M,1\leq n\leq N$ denotes the gene expression column vector of the corresponding sample $s_{m}$ on all N  features. Each sample $s_{m}$ is assigned with a label k  denoting the *k*-th subclass set $c_{k}\in C=\left\{c_{1},c_{2},\cdots \;,c_{K}\right\}$, 1≤k≤K, where K  is the total number of subclasses and $c_{k}$ is the index of the subclass with the label k , and $\left|c_{k}\right|$ represents the number of samples with the same label k .

### Flowchart of analysis

**Figure 1:**
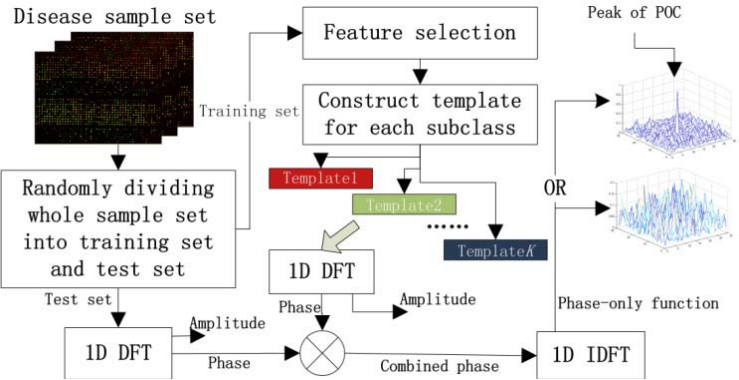
**The flowchart of applying POC analysis to identify disease types**. 1D DFT: one-Dimensional Discrete Fourier Transform; 1D IDFT: one-Dimensional Inverse Discrete Fourier Transform; Template k denotes the k-th template constructed using training set.

POC allows us evaluate the similarity of disease samples in frequency domain based on their GEPs. Figure 1 shows the flowchart of the proposed method for predicting the type of a disease sample. This method is essentially equivalent to a special case of 1NN classification method with just one virtual sample per subclass in training set. The procedure involves the following steps:

1) The entire sample set is randomly split into two disjoint parts: a training set and a test set. We then select a certain number of DEGs or differentially expressed proteins (DEPs) using the Kruskal-Wallis rank sum test (KWRST) method [37].

2) A virtual sample template for each subclass of training set is constructed by averaging all samples in the subclass. The *j-*th component of virtual sample template for subclass $c_{k}$ is $\mu_{k,j}=\left(\sum_{i\in c_{k}}x_{i,j}\right)/|c_{k}|,1\leq j\leq N$, the mean expression of the *k*-th subclass in training set for feature j . Thus the concept of virtual sample template is the same as the centroid proposed in [38].

3) The POC function is calculated between each virtual sample template and a test sample, both of which are already transformed using the one-dimensional discrete Fourier transform (1D DFT). The similarity between each virtual sample template and a given test sample is evaluated using the peak value of POC. The formalized representation of a test sample matching with all K  templates is denoted by

(1) $$r_{k}=idft\left(imag\left(poc\left(dft\left(f_{test}\right),dft\left(\mu_{k}\right)\right)\right)\right),1\leq k\leq K$$

where dft(.), poc(.), imag(.), and idft(.) denote discrete Fourier transform (DFT), POC function, taking only phase, and inverse discrete Fourier transform (IDFT), respectively. Thus the peak vector $\left(peak\left(1\right),\cdots \;,peak\left(K\right)\right)$ of the test sample $f_{test}$ matching with all K  templates can be calculated by

(2) $$peak\left(k\right)=max\left(r_{k}\right),1\leq k\leq K.$$

4) The highest peak of POC can be utilized to determine the label of the test sample $f_{test}$, that is, the label of the test sample is assigned by

(3) $$C\left(f_{test}\right)=k^{*}=arg\;max_{k}peak\left(k\right).$$

If we adopt a square matrix to represent a sample instead of the vector form of the sample, we can analyze a sample set using two-dimensional POC (2D POC) to identify disease types. The flowchart of 2D POC analysis method is very similar to the 1D POC shown in Figure 1. The only difference is that 1D DFT and 1D IDFT in Figure 1 are replaced with 2D DFT and 2D IDFT, respectively. In fact, we can easily convert a sample vector (assuming that the length of the sample vector is a square number) into a square matrix easily.

### Phase-only correlation

We adopt both 1D POC and 2D POC methods to analyze disease samples. Here we only give the mathematical description of 1D POC. The principle of 2D POC can be found in literature [28]. Given two samples $f_{a}\left(n\right)\in S$ and $f_{b}\left(n\right)\in S$, here we assume that their index ranges are n=-Q⋯Q, and T=2Q+1 for mathematical simplicity, where Q  is an integer. Let $F_{a}\left(n\right)$ and $F_{b}\left(n\right)$ denote the one-dimensional discrete Fourier transform (1D DFT) of two samples $f_{a}\left(n\right)$ and $f_{b}\left(n\right)$, respectively. They are given by

(4) $$F_{a}\left(n\right)=\sum_{l=-Q}^{Q}f_{a}\left(l\right)W_{T}^{ln}=A_{F_{a}}\left(n\right)e^{j\theta_{F_{a}}\left(n\right)}$$

(5) $$F_{b}\left(n\right)=\sum_{l=-Q}^{Q}f_{b}\left(l\right)W_{T}^{ln}=A_{F_{b}}\left(n\right)e^{j\theta_{F_{b}}\left(n\right)}$$

where n=-Q⋯Q and $W_{T}=e^{-j2\pi /T}$. $A_{F_{a}}$ and $A_{F_{b}}$ are amplitude components, and $e^{j\theta_{F_{a}}\left(n\right)}$ and $e^{j\theta_{F_{b}}\left(n\right)}$ are phase components. The cross-phase spectrum R(n) is defined as

(6) $$R\left(n\right)=\frac{F_{a}\left(n\right)\bar{F_{b}\left(n\right)}}{\left|F_{a}\left(n\right)\bar{F_{b}\left(n\right)}\right|}=e^{-j\theta \left(n\right)}$$

where $\bar{F_{b}\left(n\right)}$ denotes the complex conjugate of $F_{b}\left(n\right)$ and $\theta \left(n\right)=\theta_{F_{a}}\left(n\right)-\theta_{F_{b}}\left(n\right)$ denotes the phase difference. Only the phase information is utilized while the amplitude is discarded because phase information is significantly more important than amplitude information in preserving the properties of intrinsic pattern [27]. Thus the 1D inverse DFT (1D IDFT) of R(n) is denoted as

(7) $$r\left(n\right)=\frac{1}{T}\sum_{l=-Q}^{Q}R\left(l\right)W_{T}^{-ln}$$

where r(n) is the 1D POC function between $f_{a}\left(n\right)$ and $f_{b}\left(n\right)$, and its value has a range from 0 to 1. The correlation peak value of r(n) provides a measure of the similarity between the two samples. Usually, the larger the peak value is, the more similar the two samples are, and vice versa. The peak value decreases when the noise in a test sample and the constructed templates increase [28]. Thus high-level noise in samples may degrade the accuracy of prediction.

In contrast to the template-based POC method, we design a POC1DKNN method that utilizes 1D POC to measure the similarity of two samples and apply 5-nearest neighbor (5NN) to predict the label of test sample.

### Experimental methods

Although there is no parameter in the proposed method, the different number of pre-selected features and the different divisions of training sets and test sets can also affect the classification performance. To obtain objective results, the Balance Division Method (BDM) is used to divide each original dataset into balanced training sets and test sets [26]. For the BDM, Q  samples from each subclass of the original dataset are randomly selected and used as a training set, while the remaining samples are used as test set. For example, if we set Q  to 5 for the SRBCT dataset, then 5 samples per subclass are randomly selected, that is, 4×5=20 samples are used as a training set and the rest 83-20=63 samples are assigned to a test set. Considering that 2D POC requires the square number of features selected, we select $15^{2},\cdots \;,30^{2}$ features using KWRST to evaluate the performance of POC method because the number of genes or proteins related to complex diseases is unknown and likely different from one disease to an-other.

## Results

### Visualization of experimental results

**Figure 2:**
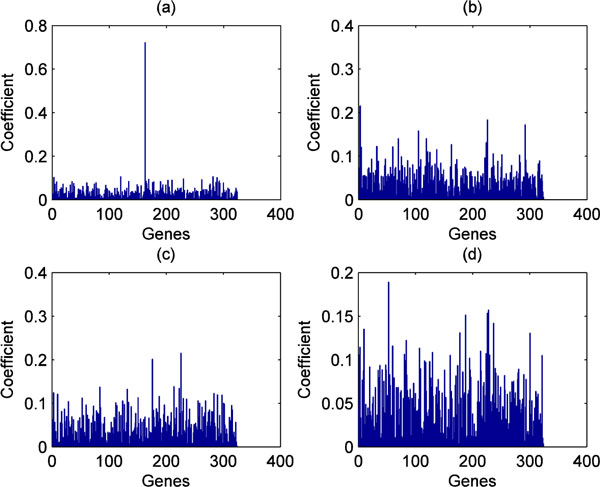
**1D cross-correlation coefficient calculated by a test sample (belonging to EWS) matching with the four templates of the SRBCT dataset, respectively**. (a) EWS subtype. (b) BL. (c) NB. (d) RMS.

**Figure 3:**
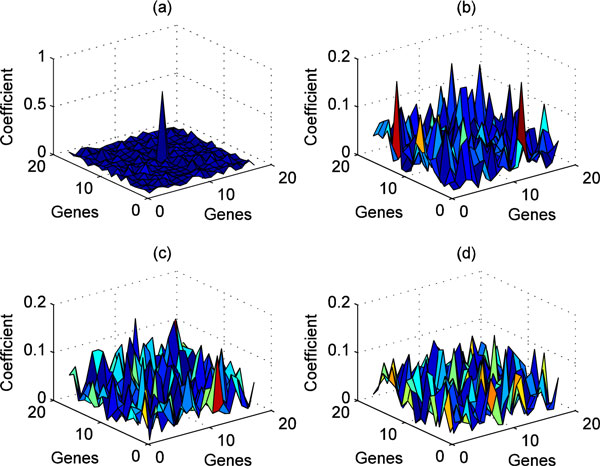
**2D cross-correlation coefficient calculated by a test sample (belonging to EWS subtype) matching with the four templates of SRBCT**. (a) EWS subtype. (b) BL. (c) NB. (d) RMS.

The results of 1D POC and 2D POC can be visually represented. Taking the SRBCT dataset as an example, 1D cross-correlation coefficients calculated by a test sample (belonging to EWS subtype) matching with the four templates corresponding to the four subtypes of the SRBCT dataset are shown in Figure 2 (a), (b), (c) and 2(d), respectively. Their matching peak values are 0.7213, 0.2153, 0.2154, and 0.1889, respectively, suggesting the test sample can be correctly assigned to EWS subtype based on these values. Figure 3 shows 2D cross-correlation coefficients calculated for a test sample (belonging to EWS subtype) compared to the four templates of the SRBCT dataset. Their matching peak values are 0.7118, 0.1971, 0.1487, and 0.1471, respectively. Thus this test sample can be easily assigned to EWS subtype.

**Figure 4:**
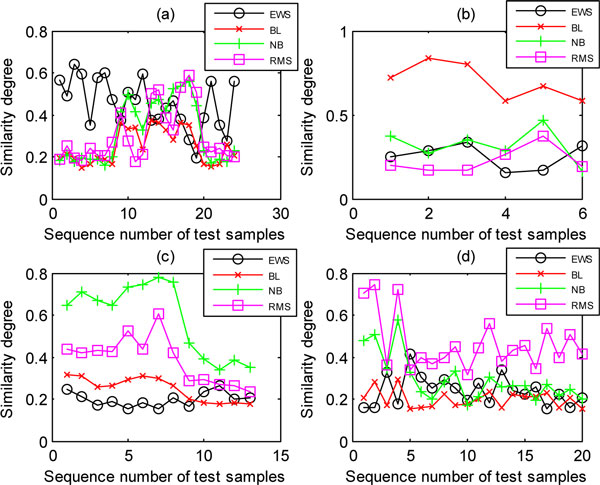
**The separability of all test samples in the SRBCT dataset**. (a) The separability of all test samples belonging to EWS subtype. (b) BL. (c) NB. (d) RMS.

The resulting separability of all test samples (belonging to the same subclass) matching with each template can be visualized using plots, from which we can visually determine which test sample is correctly or mistakenly classified. For example, Figure 4, obtained using POC based on the training set selected randomly with 5 samples per subclass from the SRBCT dataset, shows the separability of the four subtypes of the SRBCT dataset in four subplots, respectively. In each subplot, the abscissa axis denotes the sequence number of all test samples within the same subclass, and the ordinate axis denotes the similarity degree (peak value) calculated by matching test samples with each template. To make it clearer, in each subplot we connect the points belonging to the same subclass to demonstrate the separability of different subclasses. Figure 4 clearly shows that all test samples in the two subtypes (BL and NB) are correctly classified, but the classification of the EWS subtype is not perfect.

## Comparison with other methods

### Comparison with MACE method

**Figure 5:**
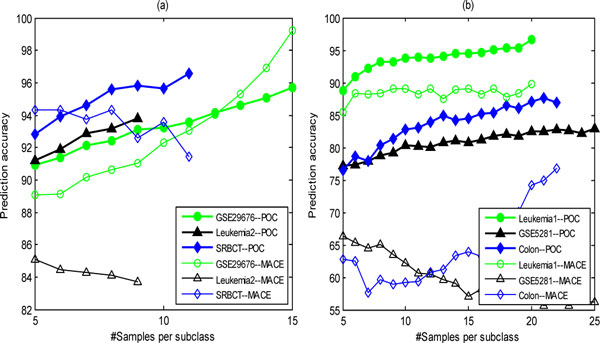
**The comparison of performance between the POC and MACE methods on the six complex disease datasets under the condition of no normalization**. (a) The performance comparison of POC and MACE on the GSE29676, Leukemia2, and SRBCT datasets; (b) The performance comparison of POC and MACE on the Leukemia1, GSE5281, and Colon datasets.

Due to the fact that the OTSDF is a method with one parameter and the performance of OTSDF and MACE are almost equivalent, for fairer comparison we do not compare POC with OTSDF in performance, while we only compare the performance of POC with the one of MACE. Like POC, MACE is also a nonparametric method that has shown good performance on recognizing tumor subtypes [26]. However, the performance of MACE is sensitive to the data scaling method used to standardize the data. POC does not require data scaling and thus it can avoid this problem. We fix the number of the selected genes to $18^{2}$ and assess the classification performance varies with regard to different sample size of training set. Figure 5 shows the comparison of performance for POC and MACE on six disease datasets, where each original dataset is divided into a balanced training set and a test set by using the BDM method with Q  varying from 5 to $\left|c_{\text{min}}\right|$, where $c_{\text{min}}=argmin_{c_{i}}\left(\left|c_{i}\right|\right),1\leq i\leq K$. If $|c_{\text{min}}|>25$, then the Q  value takes from 5 to 25. The comparison clearly shows that POC outperforms MACE in predictive accuracy on all six datasets (note that for the GSE29676 dataset only when the number of training samples is larger than 12, and MACE is obviously superior to POC in performance; for the SRBCT dataset only when the number of training samples is lesser than 7, and MACE is slightly superior to POC in performance).

### Comparison with other model-based methods

Since the template-based POC method can be used to build classifiers, we compare it with other state-of-the-art model-based classification algorithms including NBM, KNN, PNN, and SVM. For KNN, we set its k  to 5 and adopt the correlation distance (one minus the sample correlation between points) as the measure between two samples, where the correlation distance is computed by the following formula.

(8) $$d_{st}=1-\frac{\left(x_{s}-{\bar{x}}_{s}\right){\left(y_{t}-{\bar{y}}_{t}\right)}^{\prime }}{\sqrt{\left(x_{s}-{\bar{x}}_{s}\right){\left(x_{s}-{\bar{x}}_{s}\right)}^{\prime }}\sqrt{\left(y_{t}-{\bar{y}}_{t}\right){\left(y_{t}-{\bar{y}}_{t}\right)}^{\prime }}}$$

where ${\bar{x}}_{s}=\frac{1}{n}\sum_{j}x_{sj}$ and ${\bar{y}}_{t}=\frac{1}{n}\sum_{j}y_{tj}$.

For PNN, there is a smoothing parameter σ  to be tuned within the range of [01]. To determine the optimal σ  value, 5-fold cross-validation (5-fold CV) is performed by taking σ  value from 0 to 1 by step 0.1 on each training set divided randomly on original dataset using BDM. The optimal σ  is the one with the best performance of 5-fold CV. For SVM, radial basis function (RBF) kernel is used as the kernel function of SVM. There are two parameters, C  and γ , to be tuned. We use 5-fold CV on training set to determine the optimal combination of the parameters C  and γ  by screening all combinations of the following C  and γ : $C=\left\{2^{1},2^{2},\cdots \;,2^{16}\right\}$, and $\gamma =\left\{2^{-5},2^{-4},\cdots \;,2^{16}\right\}$. Because SVM requires data scaling, each dataset is standardized into one with zero mean and unit variance. Therefore, to obtain fairer comparison data scaling pre-process is performed before classification.

**Figure 6:**
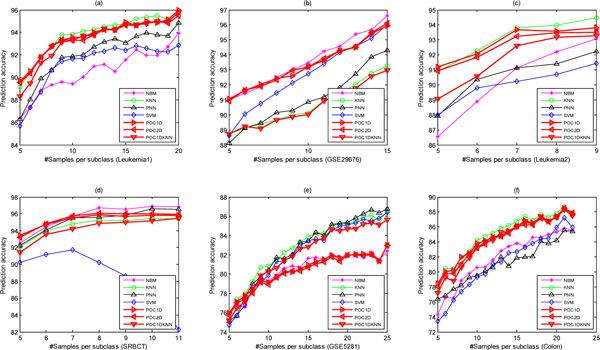
**The performance of eight methods varying with the number of training samples per subclass on the six datasets**.

Because the performance of a model is sensitive to data division into training and test sets, we repeat the procedure 200 times using randomly divided training and test sets and report the mean value of the 200 predictive accuracies for each method (Figure 6). Figure 6 shows the performance of seven methods with regard to different number of training samples per subclasses. Both POC1D and POC2D perform well and are slightly superior to POC1DKNN except on the GSE5281 dataset. Overall, our methods achieve optimal or near-optimal performance.

**Figure 7:**
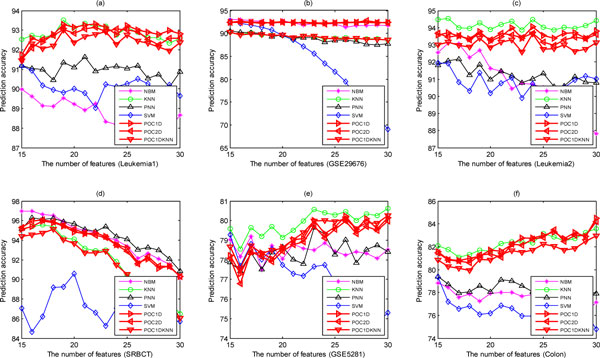
**The performance of eight methods varying with the number of features on the six datasets**.

We then fix the number of training samples per subclass to 8 but use different number of selected features and study the performance of models for each dataset (Figure 7). The results show that the performance of our method is very robust with regard to the number of features. KNN slightly outperforms our methods only on the Leukemia2 and GSE5281 datasets, but it is obviously inferior to our methods on the GSE29676 and SRBCT datasets. We have also studied the effects of using other feature filter methods such as *t*-test instead of KWRST, and the experimental results indicate that different feature filters affect less the performance of the POC-based method.

### Comparison with feature extraction-based methods

Due to the high dimensionality of dataset, feature extraction is often used to reduce the dimensionality of dataset before classification, and it plays a crucial role in simplifying classification model and improving the classification performance. Here we compare our method with five dimensionality reduction methods, i.e., PCA, LDA, ICA, LLDE, and LPP, which are extensively applied into the classification of complex disease. Our previous study suggests that the prediction accuracy depends less on classification methods [39] when the number of features extracted is small enough. Thus we also adopt the simplest classification method *k*-nearest neighbor (KNN) with correlation distance to classify disease samples, and fixedly set its k  to 5.

**Figure 8:**
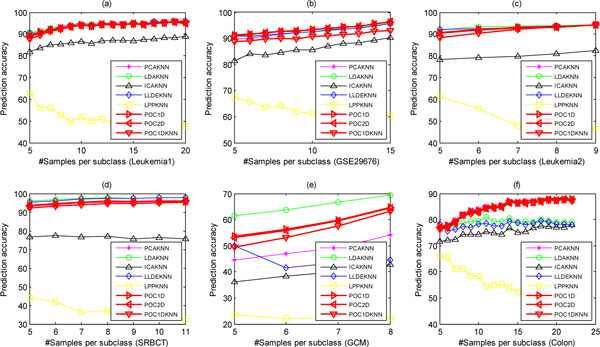
**The performance of eight methods varying with the number of training samples per subclass on the six datasets**.

**Figure 9:**
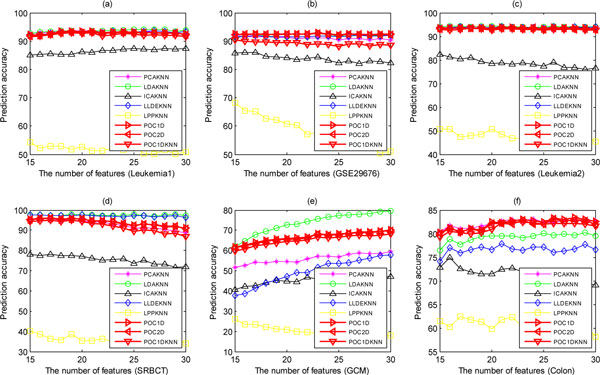
**The performance of eight methods varying with the number of features on the six datasets**.

To avoid over-fitting, before classification we extract only 5 features adopting these feature extraction methods except LDA whose number extracted is K-1. Due to these feature extraction methods require data normalization, so each dataset has been sample-wise normalized to zero mean and one variance after feature selection. We call these methods as PCAKNN, LDAKNN, ICAKNN, LLDEKNN, and LPPKNN, respectively. To further valid the effectiveness of our method on multiclass dataset, we select the GCM dataset with 14 different tumor types to evaluate the performance of our method. Figure 8 shows the performance of eight methods with regard to different number of training samples per subclasses. The results indicate that the performance of POC1D and POC2D are almost the same and slightly superior to POC1DKNN. Although LDAKNN outperforms POC on the GCM dataset, on the Colon dataset POC outperforms LDAKNN. Our method can achieve optimal or near-optimal performance and has clear biomedical meaning, compared with other five feature extraction-based methods. Furthermore, for each dataset we also fix the number of training samples per subclass to 8, and study the performance varying with the number of selected features, as shown in Figure 9, indicating that the performance of these methods is robust with regard to the number of genes and our method can also achieve the best or near-optimal performance except LDAKNN on the GCM dataset. To conclude, our novel method is very effective and can obtain the best and near-optimal performance.

### Permutation assessment

To further assess the reliability of the proposed method, we calculate the label permutation-based *p*-values [40] of the six datasets. For each dataset we fix the number of training samples in each subclass to 8 and the number of initially selected features to $18^{2}$. First we perform r=1000 randomizations of training sets and test sets, and then for each randomization we randomly permute the labels of the test set l=50 times while keeping the labels of training set original. Therefore r×l=1000×50 predictive values are obtained, which are denoted as matrix $Acc_{ij},1\leq i\leq r,1\leq j\leq l$. For each randomization, l=50 predictive values are averaged, which is denoted as $Mean\text{\_}Acc_{i},1\leq i\leq r$. The final mean can be calculated by $\sum_{i=1}^{r}Mean\text{\_}Acc_{i}/r$. p -Values can be calculated by

(9) $$p=\frac{\left|\left\{S^{\prime }\in Ŝ:Acc\left(POC,S^{\prime }\right)\geq Acc\left(POC,S\right)\right\}\right|+1}{r\times l+1},$$

where Ŝ denotes a set of r×l randomized version $S^{\prime }$ of the original dataset S and $Acc\left(POC,S^{\prime }\right)$ denotes the predictive accuracy obtained using POC on dataset $S^{\prime }$. Acc(POC,S) denotes the mean of 200 predictive accuracy using POC on 200 randomizations of training set and test set obtained on original dataset. Table 2 shows the results of permutation tests with r=1000 and l=50 using the template-based POC1D and POC2D methods on the six datasets. It is clear that the obtained classification performance is reliable because their *p*-values are very small. The mean value ${\text{B}}_{mean}$ of predictive accuracy with label permutation for each dataset is close to 1/K except the Leukemia2, SRBCT and GCM datasets (for the Leukemia2 and SRBCT datasets there is only one sample in a subclass in test set, and for the GCM dataset there are few or several samples in many subclasses in test set), where *K *is the number of subclasses in dataset, indicating that no bias occurred in the obtained results [22].

**Table 2:** Permutation tests with POC1D and POC2D on the six datasets.

	POC1D			POC2D		
	
Datasets	${\text{A}}_{mean}$	${\text{B}}_{mean}$	***p*-val**.	${\text{A}}_{mean}$	${\text{B}}_{mean}$	***p*-val**.
Leukemia1	92.79	34.64	0	92.67	34.61	0
GSE29676	92.68	23.89	0	92.70	23.90	0
Leukemia2	93.40	50.85	0	93.19	50.84	0
SRBCT	95.85	31.91	0	95.87	31.92	0
GSE5281	78.37	50.22	0	78.01	50.16	0
Colon	81.79	55.40	0.00012	81.67	55.28	0.00010
GCM	64.75	15.23	0	64.42	15.26	0

${\text{A}}_{mean}$: The mean of POC with 200 randomizations on original dataset; ${\text{B}}_{mean}$: the mean of prediction accuracy on the label permutation; and *p*-val.: the *p*-value of ${\text{A}}_{mean}$.

## Discussions

Data scaling or normalization is a very important data pre-processing step for many machine learning algorithms sensitive to the numeric ranges of attributes. There are several widely used scaling methods such as Z-score that transforms data into the one with zero-mean and one-variance, and 0-1 scaling method that transforms data into the range between 0 and 1, etc. Currently it is difficult to predict what is the best data scaling method for a given dataset [41], and no clear standard criterion can be used to evaluate various scaling methods [42]. Besides, information such as dynamic ranges might be lost during data scaling. Therefore the proposed method is advantageous over those demanding a scaling process because it does not require data scaling.

In the present study, we construct the template of each subtype using the means of the data points in the training dataset. The results demonstrate that this approach is reasonable and good performance is achieved. Nevertheless, there are certainly other ways to construct templates. For example, medians, instead of means, are another possible approach that might be more suitable for data that are not normally distributed. For the present study, we test medians but do not find significant difference from means (data not shown). Thus only the results using means are reported.

## Conclusions

A POC-based method is reported as a new technique for identifying similar gene expression signatures for the differentially expressed genes or proteins. By measuring the similarity between a test sample and the virtual sample templates constructed on training set for each subclass, the label of the test sample can be easily determined. Applying the POC-based classification method to six complex disease datasets shows that this novel method is feasible, efficient and robust. Compared with five state-of-the-art classification algorithms and five feature extraction-based methods, the proposed method can achieve optimal or near-optimal classification accuracy.

Our methods can detect the similarity of overall pattern while ignoring small mismatches between a giving test sample and templates because correlation filters are based on integration operation. Compared with the MACE and OTSDF methods, POC is not sensitive to data scaling methods. The experimental results show that the POC-based method can achieve satisfactory results even without scaling data. Moreover, there is no parameter to be tuned in POC, so this method can easily avoid the over-fitting problem as well as the effects of dimensionality curse. One possible drawback of this novel method is that high-level noise in the template can suppress the output peak. Our future work will focus on exploring novel method to construct more representative template to further improve predictive accuracy.

## Competing interests

The authors declare that they have no competing interests.

## Authors' contributions

All authors contributed to the design of the project, the interpretation of the results, and the drafting and production of the manuscript.

## Declarations

This article has been published as part of *BMC Bioinformatics *Volume 14 Supplement 8, 2013: Proceedings of the 2012 International Conference on Intelligent Computing (ICIC 2012). The full contents of the supplement are available online at http://www.biomedcentral.com/bmcbioinformatics/supplements/14/S8.
